# The impact of changing diagnostic criteria on disability in a Brazilian multiple sclerosis cohort

**DOI:** 10.1055/s-0045-1809662

**Published:** 2025-06-25

**Authors:** Felipe Toscano Lins de Menezes, Jéssica Monique Dias Alencar, Alexandre Bussinger Lopes, Leizian de Souza Amorim, Raquel Paiva Portugal, Flávia Timbó Albuquerque, Natasha Pryanca de Araújo Bessa, Larissa Sabino Ferreira Vicente, Nilton Amorim de Souza, Denis Bernardi Bichuetti, Enedina Maria Lobato de Oliveira

**Affiliations:** 1 Universidade Federal de São Paulo, Escola Paulista de Medicina, Departamento de Neurologia e Neurocirurgia, São Paulo SP, Brazil.

**Keywords:** Multiple Sclerosis, Multiple Sclerosis, Relapsing-Remitting, Prognosis, Disease Progression, Diagnosis

## Abstract

**Background:**

Updating multiple sclerosis (MS) diagnostic criteria over recent decades may have impacted disability progression.

**Objective:**

To assess the effects of the passage of time and changes in diagnostic criteria on disability.

**Methods:**

A retrospective study of Brazilian people with relapsing-remitting MS from 1994 to 2019. Descriptive analysis compared three periods based on admission: Epoch 1 (1994–2001), 2 (2002–2010), and 3 (2011–2019). Cox regressions were performed for the outcomes of the expanded disability status scale (EDSS) 6.0 and conversion to secondary progressive MS (SPMS). We compared the three Epochs in sequence, the Poser with all McDonald criteria combined, and Poser versus each McDonald criteria (2001, 2005, 2010, and 2017). A multivariate logistic regression assessed the impact of diagnostic criteria on patients reaching EDSS 6.0.

**Results:**

Time to diagnosis and treatment decreased across Epochs. For reaching EDSS 6.0, the Cox regression indicated a hazard ratio (HR) 63% lower for Epoch 3 compared with 1, an HR 50% lower for the McDonald criteria combined, and an HR 65% lower for McDonald 2010 compared with Poser. Regarding the conversion to SPMS, the HR was 53% lower for Epoch 3, 48% lower for the McDonald criteria combined, and 64% lower for McDonald 2010. The multivariate logistic regression demonstrated that incomplete recovery of initial symptoms was the main prognostic factor for reaching EDSS 6.0. However, transitioning diagnostic criteria from Poser to McDonald 2001 and 2005 decreased these odds by 54%.

**Conclusion:**

Newer diagnostic criteria have reduced the likelihood of reaching EDSS 6.0 and converting to SPMS over the past 25 years.

## INTRODUCTION

In recent decades, it has been noticed that the progression of disability is increasingly slower in people with multiple sclerosis (MS). 1 In cohorts from the pretreatment era, the median time to expanded disability status scale (EDSS) 6.0 ranged from 9 to 19 years. 2 3 4 Recent series show that this time is currently more extended; EDSS 6.0 is reached after more than 20 years of disease, 5 6 7 with some studies reporting over 30 years. 8 9 In this context, the evolution of diagnostic criteria may play a fundamental role.

Clinical information has always been the most relevant item in diagnosing MS. 10 In 1983, with Poser's criteria, we could include oligoclonal bands (OCB) and evoked potentials. 11 The McDonald 2001 criteria (Mc01) included the magnetic resonance imaging (MRI) for dissemination in space (DIS) and time (DIT). 12 The McDonald 2005 (Mc05) brought greater relevance to spinal cord injuries and shortened the MRI interval from 3 to 1 month for DIT. 13 The McDonald 2010 (Mc10) simplified the DIS definition and excluded the OCB to support it. Furthermore, Mc10 permitted DIT with two MRIs regardless of the interval between them. 14 Lastly, the McDonald 2017 (Mc17) allowed the replacement of OCB as a means of DIT. 15

A Spanish cohort demonstrated that the proportion of patients diagnosed with MS at the first clinical event progressively increased from 25.2% with Poser to 55.1% with Mc17. 16 Each update increases diagnostic sensitivity and allows for early detection and treatment. However, the impact of changing criteria on disability in Brazilian patients remains uncertain.

The present study aims to delve into a Brazilian cohort to understand the impact of early diagnosis and treatment on the evolution of disability. Specifically, we strive to unravel the effect of the changing criteria on reaching EDSS 6.0 and converting to secondary progressive multiple sclerosis (SPMS).

## METHODS

We conducted an observational study of a retrospective cohort evaluating medical records from the Neuroimmunology Clinic at Hospital São Paulo, Universidade Federal de São Paulo, part of the Brazilian Public Health System. The study was approved by the Ethics Committee at Universidade Federal de São Paulo (CAAE: 40467720.8.1001.5505). We obtained written informed consent from most of the patients. The Ethics Committee allowed data collection from those lost to follow-up.

### Patient selection

We included patients admitted between February 1, 1994, and June 30, 2019, aged 18-years or older, diagnosed with MS per current diagnostic criteria, 11 12 13 14 15 with at least three visits and 6 months of follow-up. Exclusion criteria were pediatric MS, progressive phenotypes at the beginning of follow-up, and incomplete records. Data collection continued through the last visit, up to December 2020.

### Definitions

We define Epochs as a period within 25 years in which the first appointment occurred. Epoch 1 was between 1994 and 2001, when the Poser criteria were the default for MS diagnosis. Epoch 2 was from 2002 to 2010, equivalent to the primary use of Mc01 and Mc05. Finally, Epoch 3 was between 2011 and 2019, corresponding to the primary use of Mc10 and Mc17. For further analysis, patients were also classified into five categories according to the criteria for establishing their diagnosis: Poser, and Mc01, 05, 10, and 17.

Relapse was defined as new or worsened symptoms consistent with a demyelinating syndrome lasting at least 24 hours, without fever or infection. 15 The global annualized relapse rate was calculated as the number of relapses by total disease duration. Incomplete recovery of the initial symptoms was defined as a residual and sustained deficit of at least 2 points in one of the classes, according to the Kurtzke functional system of EDSS. 17 18

For disease-modifying therapy (DMT), all variables pertain to treatments used for at least 1 year. To assess treatment evolution over the years, we conducted a flow analysis categorizing therapies for each Epoch into seven classifications:

- Low-dose interferons (IFN-β-1a 30µg, IFN-β-1a 22µg, any sub-doses of IFN-β-1a 44µg or IFN-β-1b 250μg);
- High-dose interferons (IFN-β-1b 44μg, IFN-β-1b 250μg);
- Glatiramer acetate;
- Oral immunosuppressors (azathioprine, methotrexate) alone or with interferons;
- Oral DMT (teriflunomide, dimethyl fumarate, fingolimod);
- Natalizumab; and
- Intravenous immunosuppressors (ocrelizumab, rituximab, mitoxantrone, cyclophosphamide) or bone marrow transplantation (BMT).

Additionally, we classified the first DMT into two groups based on relapse reduction: low efficacy (interferons, glatiramer acetate, teriflunomide, azathioprine, and methotrexate) and moderate-to-high efficacy (dimethyl fumarate, fingolimod, natalizumab, and cyclophosphamide). 19

The EDSS 6.0 is defined as unilateral gait support becoming necessary for 100 m. 17 Conversion to SPMS was determined based on clinical documentation in the medical records, provided it met the following objective criteria: an irreversible increase of at least one point in the scale within 1-year without relapses, and a minimum rate of 4.0. 20 21 Patients with final EDSS 6.0 or higher, without documented SPMS diagnosis, were classified as having SPMS if they fulfilled the above criteria. The conversion date was recorded when EDSS 6.0 was reached. Our study had 22 patients with this rate, with a minimum time to SPMS of 1.8 years.

### Statistical analysis

For descriptive analysis, we compared three groups according to the Epoch at first appointment. Categorical data were presented as absolute values (n) and relative frequencies (%). Percentages correspond to each variable's available data. Due to non-normal distribution, continuous variables were described using median and interquartile range (IQR). The chi-squared and Kruskal-Wallis' tests compared qualitative and quantitative variables.

We constructed a Cox regression for two independent outcomes: reaching the EDSS 6.0 and conversion to SPMS. First, the survival analyses were performed to compare the three Epochs found during the first appointment. Afterward, we compared a group diagnosed with Poser's and another diagnosed with McDonald's combined criteria (Mc01, Mc05, Mc10, and Mc17). This separation marks the introduction of MRI into the workflow, starting with Mc01. Furthermore, we conducted a third analysis measuring the impact of changing the five diagnostic criteria for the two endpoints. To minimize the effect of disease duration on outcomes, we included it as an independent variable in each survival curve.

Finally, we performed a multivariate logistic regression for the dependent variable EDSS 6.0, to assess the effect of different diagnostic criteria along with other clinical factors. Due to the difference in the number of patients, it was necessary to reorganize the diagnostic criteria into three groups: Poser, Mc01 and 05, and Mc10 and 17. The other independent variables were clinical characteristics that can interfere with the outcome according to natural history and prognostic studies: incomplete recovery of initial symptoms, motor and multi-topographic initial symptoms, number of relapses before the first appointment, during the 2 and 5 initial years of disease, and the global annualized relapse rate. For this analysis, initially, we did an exploratory study of the chosen variables, and after, we identified the best model through the backyard method.

Significance was set at *p* < 0.05. Statistical analysis was performed using the R software (R Foundation for Statistical Computing), version 4.0.5.

## RESULTS

**Figure 1:**
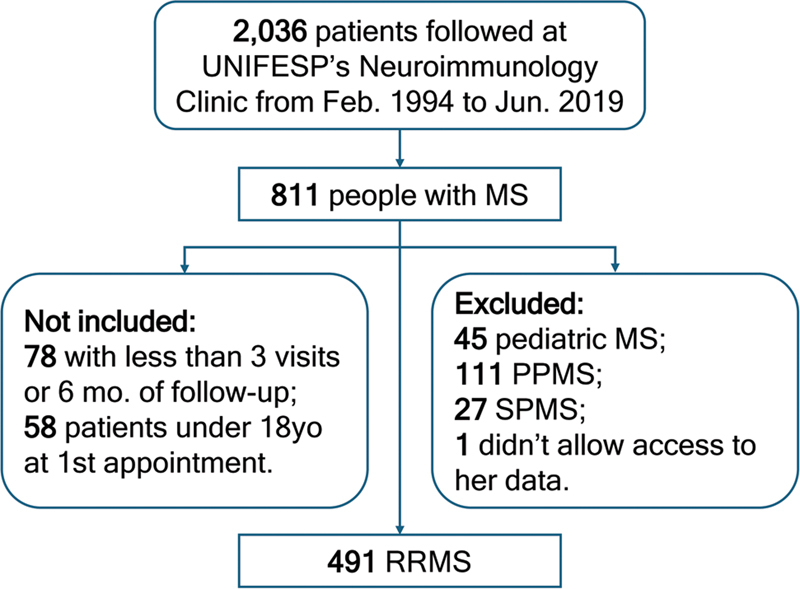
Abbreviations: mo., months; MS, multiple sclerosis; PPMS, primary progressive multiple sclerosis; SPMS, secondary progressive multiple sclerosis; UNIFESP, Universidade Federal de São Paulo; yo, years old. Flowchart of patient selection.

We retrieved 811 people with MS from 2,036 medical records. According to the inclusion and exclusion criteria, our study had a total of 491 patients with RRMS from a single center ( Figure 1 ). They were mainly women (76%) and White (71.1%), with a median follow-up of 8.1 years. Table 1 presents the cohort characteristics and compares them across the three Epochs of the initial follow-up.

**Table 1:** Patients' characteristics according to Epochs at the first appointment ^a^

Measure		Total ( *N* = 491)	Epoch 1 (1994–2001) ( *N* = 81)	Epoch 2 (2002–2010) ( *N* = 207)	Epoch 3 (2011–2019) ( *N* = 203)	*p* -value
General features	Woman, n (%)	373 (76.0)	61 (75.3)	156 (75.4)	156 (76.8)	0.929
	White, n (%)	347/488* (71.1)	60 (74.1)	153 (73.9)	134/200* (67.0)	0.249
	Age at onset, y, m (IQR)	28.8 (23.8; 35.3)	28.0 (23.8; 39.9)	30.0 (24.4; 35.7)	28.2 (23.7; 34.2)	0.233
	Time for the first appointment, y, m (IQR)	2.0 (0.5; 5.0)	2.2 (1.0; 6.8)	2.1 (0.7; 4.7)	1.5 (0.4; 4.5)	0.010
	Time to MS diagnosis, y, m (IQR)	2.5 (1.0; 6.0)	2.4 (1.2; 6.8)	3.0 (1.1; 6.6)	2.0 (0.7; 5.1)	0.022
	Disease duration, y, m (IQR)	12.2 (7.4; 18.0)	20.3 (13.7; 25.3)	14.6 (11.4; 18.3)	7.8 (4.8; 10.1)	< 0.001
	Follow-up duration	8.1 (4.4; 13.1)	16.3 (7.6; 20.6)	12.0 (9.2; 14.4)	5.1 (3.0; 7.2)	< 0.001
Diagnostic criteria, n (%)	Poser	72 (14.7)	72 (88.9)	−	−	−
	McDonald					
	2001	82 (16.7)	8 (9.9)	74 (35.7)	−	−
	2005	117 (23.8)	−	111 (53.6)	6 (3.0)	−
	2010	188 (38.3)	1 (1.2)	20 (9.7)	167 (82.3)	−
	2017	32 (6.5)	−	2 (1.0)	30 (14.8)	−
Clinical features	Initial symptoms, n (%)					
	Motor	203/488* (41.6)	39 (48.1)	92/204* (45.1)	72 (35.5)	0.061
	Multitopographic	43/488* (8.8)	5 (6.2)	20/204* (9.8)	18 (8.9)	0.621
	Incomplete recovery	95/473* (20.1)	16/73* (21.9)	39/198* (19.7)	40/202* (19.8)	0.913
	Number of relapses, m (min-max)					
	Before first appointment	2 (1–19)	3 (1–10)	2 (1–9)	2 (1–19)	< 0.001
	2 years of disease	2 (1–9)	2 (1–9)	2 (1–7)	2 (1–6)	0.013
	5 years of disease	3 (1–13)	3 (1–13)	3 (1–12)	2 (1–13)	0.002
	Global ARR, m (IQR)	0.4 (0.2; 0.7)	0.4 (0.3; 0.7)	0.3 (0.2; 0.5)	0.5 (0.2; 0.8)	0.002
	Initial EDSS, m (IQR)	2.0 (1.0; 3.0)	2.0 (1.0; 3.0)	2.0 (1.0; 2.5)	2.0 (1.0; 2.5)	0.150
	Final EDSS, m (IQR)	2.5 (1.5; 6.0)	6.0 (2.5; 7.0)	3.0 (2.0; 6.0)	2.0 (1.0; 3.0)	< 0.001
Treatment	Received DMT at least 1 year, n (%)	427 (87.0)	73 (90.1)	190 (91.8)	164 (80.8)	0.003
	Time to first DMT, y, m (IQR)	2.6 (1.0; 6.1)	2.8 (1.4; 7.2)	3.0 (1.1; 6.5)	2.1 (0.8; 4.8)	0.020
	Age at first DMT, y, m (IQR)	33.7 (27.3; 42.1)	33.4 (28.4; 44.4)	34.3 (27.8; 42.7)	32.2 (27.0; 38.3)	0.117

Abbreviations: ARR, annualized relapse rate; DMT, disease -modifying therapies; EDSS, expanded disability status scale; IQR, interquartile range; m, median; min, minimum; max, maximum; MS, multiple sclerosis; n, number; y, years. Notes: The Kruskal-Wallis test was used for quantitative variables, and the chi-squared test for qualitative variables. A *p* -value < 0.05 was considered statistically significant differences. The diagnosis criteria are presented without statistical tests. ^a^ Categorical data are presented as number (percentage of total). *When only incomplete data were available, number/total available (percentage based on available information for each subject).

Patients starting follow-up in Epoch 1 ( *n* = 81) were diagnosed using the Poser criteria. Epoch 2 ( *n* = 207) comprises patients diagnosed with the Mc01 and 05, while those from Epoch 3 ( *n* = 203) were diagnosed primarily with the Mc10 and 17. It is worth highlighting that over Epochs, the first visit to an MS specialist and time to diagnosis occurred within shorter periods. We found fewer relapses before the first appointment and during the initial 5 years of disease across Epochs. These findings are connected to the reduction in the time to the first DMT, which decreased from 3 years in Epochs 1 and 2 to 2.1 years in Epoch 3.

**Figure 2:**
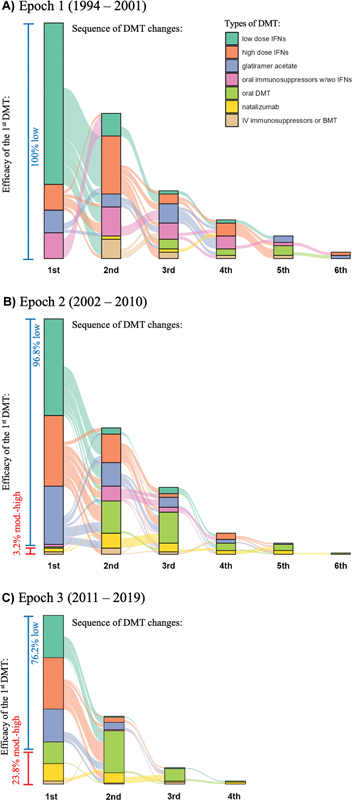
Abbreviations: BMT, bone marrow transplant; DMT, disease-modifying therapy; IFNs, interferons; IV, intravenous; mod-high, moderate-to-high; w/wo, with or without. Notes: The bars in assorted colors represent the categories of DMT, as explained in the color key in the upper right corner. Patients who did not change categories or stop using any DMT were no longer counted in subsequent bars. The frequency of low- and moderate-to-high-efficacy DMTs is indicated to the left of the first bar from each Epoch. Diagram illustrating the sequences of DMT usage from the inception of Epochs **(A)** 1; **(B)** 2; and **(C)** 3.

Epoch 3 had the highest percentage of patients starting DMT before a second relapse, with Epoch 1 at 2.7%, 2 at 17.9%, and 3 at 24.4%. Among those starting treatment within five years, the figures were 64.4% for Epoch 1, 70.5% for 2, and 75.6% for 3. For treatment before reaching EDSS 3.0, Epoch 1 had 64.4%, 2 had 78.9%, and 3 had 84.1%. Variations in therapy selections were noted across Epochs ( Figure 2 ). In Epoch 1, low- and high-dose interferons and glatiramer acetate were predominant in every stage. By Epoch 2, while those drugs remained central, more second-line options emerged, particularly oral DMT and natalizumab. In Epoch 3, oral DMT and natalizumab emerged as first-line options and dominated the subsequent choices. The use of moderate-to-high efficacy DMT as first-line therapy increased from 0% in Epoch 1 to 23.8% in 3.

### Cox regressions

According to survival analysis for the entire cohort, the median time to reach EDSS 6.0 was 22.5 years, with a 95% confidence interval (CI) of 20.9 to 26.5, while the median time of conversion to SPMS was 20.9 years (95% CI: 19.1–24.7). All the Cox regression analyses are presented in Table 2 .

**Table 2:** Cox regression results for EDSS 6.0 and SPMS outcomes

		EDSS 6.0		SPMS	
Variable		HR (95% CI) ^a^	*p* -value	HR (95% CI) ^a^	*p* -value
Epochs comparison	2 (2002–2010) ^b^	0.63 (0.42; 0.97)	0.035	0.92 (0.63; 1.35)	0.684
	3 (2011–2019) ^b^	0.37 (0.20; 0.65)	0.001	0.47 (0.28; 0.81)	0.006
	Disease duration (years)	0.92 (0.89; 0.96)	< 0.001	0.33 (0.19; 0.57)	< 0.001
Poser versus McDonald combined	McDonald combined ^c^	0.50 (0.33; 0.76)	0.010	0.52 (0.35; 0.78)	0.010
	Disease duration (years)	0.93 (0.90; 0.96)	< 0.001	0.92 (0.89; 0.95)	< 0.001
Each McDonald criteria comparison	2001 ^c^	0.61 (0.37; 0.98)	0.043	0.64 (0.40; 1.01)	0.054
	2005 ^c^	0.52 (0.31; 0.88)	0.015	0.58 (0.36; 0.93)	0.024
	2010 ^c^	0.35 (0.20; 0.62)	< 0.001	0.36 (0.21; 0.60)	< 0.001
	2017 ^c^	0.13 (0.02; 0.95)	0.045	0.11 (0.02; 0.85)	0.034
	Disease duration (years)	0.92 (0.88; 0.95)	< 0.001	0.91 (0.88; 0.94)	< 0.001

Abbreviations: EDSS, expanded disability status scale; HR, hazard ratio; SPMS, secondary progressive multiple sclerosis. Notes: Central columns show EDSS 6 results; the right columns display SPMS results. ^a^ Adjusted hazard ratio; ^b^ reference by the Epoch 1 (1994–2001) category; ^c^ reference by the Poser criteria category.

**Figure 3:**
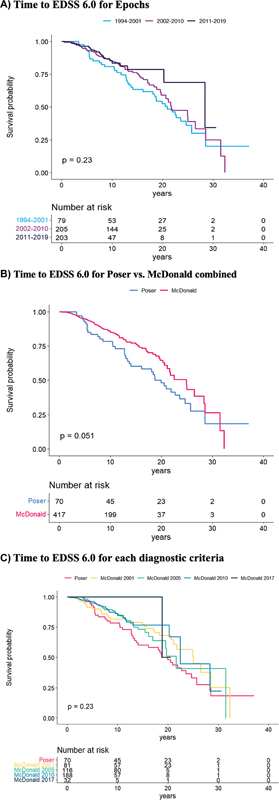
Abbreviation: EDSS, expanded disability status scale. Survival curves for time to reach EDSS 6.0 are compared across **(A)** Epochs; **(B)** Poser versus McDonald criteria; **(C)** all five criteria (Poser, as well as McDonald 2001, 2005, 2010, and 2017).

**Figure 4:**
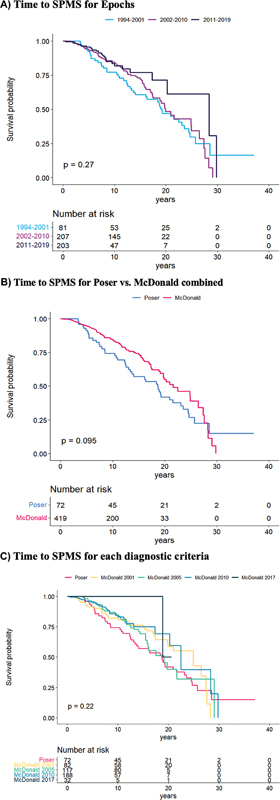
Abbreviation: SPMS, secondary progressive multiple sclerosis. Survival curves for time to conversion to SPMS are compared across **(A)** Epochs; **(B)** Poser versus McDonald criteria; **(C)** all five criteria (Poser, as well as McDonald 2001, 2005, 2010, and 2017).

In the multiple Cox regression comparing Epochs and adjusting for disease duration, the adjusted hazard ratio (aHR) for reaching EDSS 6.0 was 47% lower for Epoch 2 (aHR: 0.63, 95% CI: 0.42–0.97) compared with Epoch 1, and 63% lower for Epoch 3 (aHR: 0.37, 95% CI: 0.20–0.65), as shown in Figure 3A . Epoch 3 reduced the risk of converting to SPMS by 53% (aHR: 0.47, 95% CI: 0.28–0.81) when compared with 1, while 2 did not yield statistically significant results ( Figure 4A ).

A similar Cox analysis, comparing the combined McDonald with the Poser criteria, found a 50% lower HR for reaching EDSS 6.0 (aHR: 0.50, 95% CI: 0.33–0.76), as shown in Figure 3B . Furthermore, we found a 48% lower HR for converting to SPMS (aHR: 0.52, 95% CI 0.35–0.78), as shown in Figure 4B .

The five diagnostic criteria were compared using Cox regression, accounting for disease duration. The results revealed that newer diagnostic criteria were associated with lower hazard ratios for reaching EDSS 6.0 and transitioning to SPMS. The key finding was that the Mc10, compared with Poser, reduced in 65% the risk of reaching EDSS 6.0 (aHR: 0.35, 95% CI: 0.20–0.62), as shown in Figure 3C , and in 64% the HR of converting to SPMS (aHR: 0.34, 95% CI: 0.21–0.60). Additionally, the Mc17 showed an 87% lower HR of reaching EDSS 6.0 (aHR: 0.13, 95% CI: 0.02–0.95), and 89% lower HR of converting to SPMS (aHR: 0.11, 95% CI: 0.02–0.85), as shown in Figure 4C .

### Logistic regression

To analyze the effect of changing diagnostic criteria through logistic regression, we grouped patients according to the diagnostic criteria used: Poser ( *n* = 72), Mc01 and 05 ( *n* = 199), and Mc10 and 17 ( *n* = 220).

The multivariate logistic regression indicated that incomplete recovery of the initial symptoms was the most significant prognostic factor for reaching EDSS 6.0, with an adjusted odds ratio (aOR) of 4.46 (95% CI: 2.41–8.36). The number of relapses before the first appointment and during the first 5 years were also predictors of poor prognosis.

**Figure 5:**
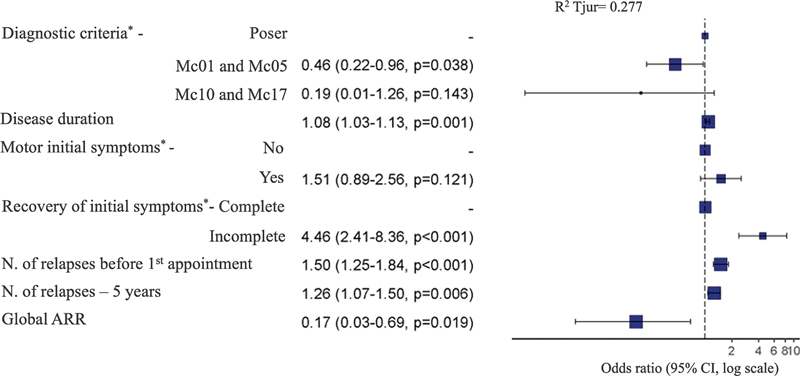
Abbreviations: ARR, annualized relapse rate; Mc01, McDonald 2001; Mc05, McDonald 2005; Mc10, McDonald 2010; Mc17, McDonald 2017; N, number. Note: *Categorical variables. Diagram of the multivariate logistic regression for the endpoint EDSS 6.0.

Conversely, the global annualized relapse rate was the best prognostic factor for not reaching EDSS 6.0 with an aOR of 0.17 (95% CI: 0.03–0.69). This variable is a ratio with disease duration as its denominator; therefore, patients with shorter disease duration could lead to misinterpretation of this finding. However, the disease duration as an independent variable did not impact the outcome. Finally, in this multivariate analysis, the Mc01 and Mc05 were protectors compared with Poser, with 54% lower odds of reaching EDSS 6.0 (aOR: 0.46, 95% CI: 0.22–0.96), otherwise changing to Mc10 and 17 had no significance ( Figure 5 ).

## DISCUSSION

We presented a Brazilian cohort with a median time to EDSS 6.0 exceeding 20 years and more than 80% of the population treated, similar to other cohorts in the posttreatment era. 5 6 7 8 9 In this retrospective study of the previous 25-years, the disability prognosis of people with MS improved over time.

Numerous international studies have demonstrated a slower progression to EDSS 6.0. A Swedish cohort of 7,331 patients diagnosed with MS between 1995 and 2010 indicated that the risk of reaching EDSS 6.0 decreased at a rate of 7% per year. 22 Multiple factors may explain this improvement in prognosis, including epidemiologic and environmental factors, such as stopping smoking, healthier diets, and vitamin D intake. Otherwise, earlier diagnosis and treatment may have a significant effect. 1 23

A Barcelona cohort monitored patients since their clinically isolated syndrome and demonstrated a 77% reduction in diagnosis delay when using Mc17 compared with the Poser criteria. Their median time to MS diagnosis dropped from 20 months during the Poser period (1994–2000) to 4.6 months in the Mc17 period (2017–2020). 16 In Denmark, the time to diagnosis fell from 12 (1996–2000) to 8.2 months (2016–2020). 24 Over the past decade, the time required for diagnosis has varied across populations, even among developed countries with similar MS prevalence rates. Between 2014 and 2019, Germany's median diagnosis time was 3 months, the United Kingdom's was 19 months, and the United States's was 60 months. 25 In developing nations, delays in diagnosis may be worsened by factors such as a lack of awareness about the disease, a shortage of healthcare providers, including neurologists and MS specialists, and difficulties in accessing diagnostic tests. 26 27

The time to diagnosis can differ even within the same country. A study of 2,974 Brazilians with MS found an average diagnostic delay of 3.65 years over two decades, with regional variations ranging from 1.4 years in the Midwest to 5.9 years in the Northeast. Although the latter region reported more progressive cases, this data highlights disparities in healthcare access across our regions. 28 Another Brazilian study found that patients with health insurance received a final diagnosis within 0.5 years, while those without it faced a delay of 4.9 years. 29 Between 2010 and 2019, we observed a median time to diagnosis of 2 years for patients relying on the Brazilian public healthcare system. This may significantly contribute to the longer diagnostic timelines for MS in our country compared with current global trends. However, our findings indicate that diagnosis and, notably, the initiation of treatment for Brazilians have occurred earlier over the decades.

The selection of therapies has changed across Epochs, with moderate-to-high efficacy DMTs increasingly used as first-line treatments. In Denmark, patients receiving these therapies within 12 months of diagnosis rose from 1.1% (1996–2000) to 17.6% (2016–2020). 24 From 2014 to 2019, moderate-to-high efficacy DMTs were the first line for 31.7% of cases in the United Kingdom, 20.6% in the United States, and 17% in Germany. 25 Changes in treatment paradigms could also influence prognosis, as it has been demonstrated that fingolimod, alemtuzumab, or natalizumab can reduce the risk of SPMS by 44% when used as first-line therapies, compared with glatiramer acetate or interferons. 30 We acknowledge the exclusion of the treatment variable from our analysis, as we believe the topic warrants a separate and detailed investigation. However, regardless of treatment advances, revised diagnostic criteria are valuable for anticipating diagnosis and treatment. In logistic regression, they influenced disability independently of traditionally poor prognostic factors.

Regarding the EDSS 6.0, we found a 47% lower risk of reaching this threshold in Epoch 2, consistent with the results from Mc01 (39%) and Mc05 (48%). During Epoch 3, the likelihood of reaching EDSS 6.0 decreased by 63%, similar to Mc10 (65%). The most recent criteria, Mc17, demonstrated better outcomes in avoiding EDSS 6.0. However, these results may be limited by the small sample size and short follow-up periods. A comparison of all McDonald's criteria combined versus Poser showed a 50% lower risk of reaching EDSS 6.0. Our multivariate logistic regression also demonstrated a similar impact of changing the criteria from Poser to Mc01 and 05. Indeed, this can be explained by the use of MRI to diagnose subtler cases and start treatment earlier. 31 32

The Barcelona cohort assessed the risk of reaching EDSS 3.0 and identified differences based on diagnostic criteria. The HR reductions were 53% for Mc01, 75% for Mc05, 70% for Mc10, and 93% for Mc17. These prognostic improvements persisted even after accounting for potential biases introduced by the Will Rogers phenomenon, due to changes in diagnostic criteria over time. 16 33 To address this effect, we adjusted the model for disease duration.

The risk-reducing impact regarding the conversion to SPMS grew as McDonald's criteria and Epochs evolved. Due to numerous yet distinct definitions, we must approach these conversion studies cautiously. 34 Nonetheless, our findings align with international studies, which indicate a gradual decline in the likelihood of SPMS conversion over the years, even in the treatment era. This is demonstrated by a large Italian cohort that revealed a 42% reduction in SPMS incidence between the periods of 1993 to 1997 and 2014 to 2018. 35

There are intrinsic limitations to retrospective analysis. Although data were registered prospectively, it is impossible to rule out physician cognitive bias while evaluating disease severity, unmasked neurological examinations, nonuniform data entry even within a team, and missing data. Although São Paulo is a metropolis with a diverse population and descendants of various nationalities, our findings cannot be generalized to other countries. However, our results align with recent studies from North America and Europe.

In conclusion, our findings extend up to Mc17, highlighting a reduction in the risk of reaching EDSS 6.0 and transitioning to SPMS over the last 25 years. Revising diagnostic criteria has enhanced disability prevention by facilitating earlier diagnosis and RRMS treatment in Brazil. In 2024, a panel updated McDonald's criteria, with the revised version expected to be published in 2025. Continued research will be crucial to determine whether these updates lead to better disability outcomes. Prospective studies are necessary to explore the influence of numerous factors on the natural history of the disease.
